# High‐throughput sequencing reveals the change of TCR α chain CDR3 with Takayasu arteritis

**DOI:** 10.1002/iid3.1122

**Published:** 2023-12-22

**Authors:** Bowen Zha, Lili Pan, Na Gao

**Affiliations:** ^1^ Department of Education Beijing Anzhen Hospital, Capital Medical University Beijing China; ^2^ Department of Rheumatology Beijing Anzhen Hospital, Capital Medical University Beijing China

**Keywords:** complementarity‐determining region 3, high‐throughput sequencing, T cell receptor, Takayasu arteritis

## Abstract

**Objective:**

Takayasu arteritis (TAK) is an inflammatory disease of blood vessels, and its pathogenesis is not clear at present. In this study, we explored the immunological characteristics of T cell receptor (TCR) α‐chain complementarity‐determining region 3 (CDR3) in patients with TAK.

**Methods:**

Five untreated patients with TAK were collected from June 2019 to December 2019. Four healthy blood samples were matched as the control group. The blood mononuclear cells were separated, and RNA was extracted for reverse transcription to obtain complementary DNA. Then high‐throughput sequencing was performed. The quality of samples was evaluated by principal component analysis. We compared the diversity and expression of TCR α‐chain between TAK group and control group. R software was used for statistical analysis and drawing, and Mann–Whitney *U* test was used to analyze the differences between the two groups.

**Results:**

The results showed that there was a significant difference in the diversity of TCR α‐chain CDR3 between the two groups. Three V region genes expression significantly higher in the TAK patients than in the control group. A total of 196 VJ rearrangement genes are significantly different between the two groups, of which 149 rearrangement genes in the TAK group are lower than those in the control group, and 47 rearrangement genes in the TAK group are higher than those in the control group.

**Conclusion:**

Patients with TAK have a unique TCR α‐chain CDR3 library. These characteristic genes may be a marker for early diagnosis and provide a new theoretical basis for treating TAK.

## Graphical abstract

Frequency chart of VJ rearrangement gene in TAK group and control group

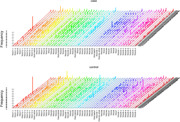

## 1 BACKGROUND

Takayasu arteritis (TAK) is a kind of vasculitis with an unknown mechanism, which mainly accumulates in the aorta and its proximal branches.^1^ It is believed that the disease can occur in all races, but most of them are young women in Asia.^2^ The current epidemiological survey shows that the incidence rate is about 2–3/million people in Korea, 2.3/million in Kuwait, and 0.4–1.3/million in Europe.^3^, ^4^, ^5^ A study in India shows that 7.2% of patients always find it difficult to recover, significantly affecting their lives.^6^, ^7^

Existing study prove that T cells are closely related to the occurrence and development of TAK. T cell receptor (TCR) is composed of α chain and β chain. Alpha chain, also known as light chain, is encoded by V, D, and J gene segments. Alpha chain is divided into the constant region and variable region, among which the most important variable region is complementarity‐determining region 3 (CDR3), which plays an important role in T cell recognition.^8^ Current research finds that after the onset of TAK, Th1 cells, and Th17 cells will have inflammatory infiltration, gather on the blood vessel wall, and release cytokines such as interferon to trigger systemic reaction.^9^ The traditional drugs for treating TAK are glucocorticoids and other antirheumatic drugs, mainly aiming at cytokines produced by Th1 cells.^10^ Although it has a good curative effect, it also brings side effects to patients. Moreover, in the face of some refractory patients, the above drugs cannot play a role.^11^

In recent years, progress has been made in studying the pathogenesis of TAK, which has led to the development of targeted biotherapy. Among them, IL‐6 and tumor necrosis factor‐α (TNF‐α) are currently considered promising therapeutic targe.^12^, ^13^ In addition, new insights into Janus kinase (JAK) inhibitors, rituximab, and other drugs have been reported continuously.^14^, ^15^, ^16^

In this study, the change of T cell α chain CDR3 with TAK was studied by high‐throughput sequencing to provide new theoretical ideas for the pathogenesis, diagnosis, and treatment of TAK.

## 2 MATERIALS AND METHODS

### 2.1 Patients and controls

Five newly diagnosed patients fulfilled the criteria for the classification of TAK developed by the American College of Rheumatology in 1990 in Beijing Anzhen Hospital were collected. The age, sex, disease type, erythrocyte sedimentation rate, C reactive protein, level of IL‐6 and TNF‐α, National institutes of health score, and the Indian Takayasu clinical activity (ITAS‐A) score were collected. Four age and sex‐matched healthy volunteers were enrolled as controls. The Medical Ethics Committee of Beijing Anzhen Hospital approved this study (Approval number: 20220.39X).

### 2.2 T cell separation and RNA extraction

A total of 10 mL of peripheral venous blood from patients with TAK and healthy controls were collected. T cells were isolated by using specific monoclonal antibodies of T cells of magnetic polystyrene beads. After separation, the RNA of T cell was extracted by Omega's nucleic acid purification kit, stored in TE buffer, and placed in a pollution‐free container. The nozzle was closed and labeled. Then reverse transcribe RNA into complementary DNA by reverse transcription kit.

### 2.3 Multiplex PCR amplification and high‐throughput sequencing

PCR reaction system was established by using TCR α chain amplification primers, and semi‐quantitative amplification of TCR α chain CDR3 was realized by multiplex PCR. Using Illumina's official software BCF 2 fastq (Version 2.15.0.4), according to the sample index sequence, the binary BCF format file of Illumina sequencer was converted and split into a single sample readable file FastQ format. Cutadapt (version 1.16) was used to remove sequencing and anti‐pollution linker sequences, and low‐quality bases were deleted to generate clean reads. Use mixer software to compare clean reads with the IMGT database to generate a vdj file. And compare the clone assembly of files to generate the final clone file.

### 2.4 Biological information analysis

The redundant sequences, short sequences, and sequences with poor sequencing quality in the data were removed by IRmap, a bioinformatics software of IR Company in the United States. The data were compared with the public database IMGT query. The immunological characteristics of samples were described by D50, shannon entropy, principal component analysis (PCA), gene frequency analysis, V region gene distribution, and VJ gene rearrangement difference.

D50 is an index reflecting the level of diversity, defined as the minimum percentage of different CDR3 that accounts for at least half of the total CDR3 in the cell group or subgroup of the immune system.^17^ Sequencing after expanding the immune group library, sorting out the expanded immune group library, and sequencing results. According to the expression level from high to low, then add it from high to low, and when it is added to 50%, calculate how many different CDR3 series are included, which is called the number of cell clones. The percentage of the number of clones in the total is the D50 value. The higher the D50 value, the higher the clone diversity. Shannon entropy can reflect the diversity of the community. It is used to measure the uncertainty of variables. The greater the uncertainty of variables, the higher Shannon entropy.

PCA is widely used to reduce the high dimension. Its main principle is to project high‐dimensional data into low‐dimensional space, extract the main factors of things and reveal their essence.^18^ In the PCA diagram, if the samples are clustered together, they have little difference and low heterogeneity. If the distance is far, these samples have great differences and high heterogeneity.

### 2.5 Statistical analysis

R was used for statistical inspection and drawing. The measurement data conforming to normal distribution were expressed by mean and standard deviation, and the comparison between groups adopts a two‐sample *t*‐test. The measurement data that do not conform to normal distribution use median and quartile to represent data, and the Mann–Whitney *U* test was used to compare the two groups. Test standard *p* < .05 is statistically significant.

## 3 RESULTS

### 3.1 Clinical characteristics of TAK patients

There were four female and one male patient in this study, and the age was 27–45 years old. The ITAS‐A scores were all below 5 points. Four patients had the angiographic classification pattern of Type V (Table 1).

**Table 1:** General basic information of TAK group.

No	Age (years)	Sex	Type of TAK	ESR mm/1 h	CRP mg/L	IL‐6 pg/mL	TNF‐α pg/mL	NIH score	ITAS‐A score
1	28	Female	V	5	0.35	2.3	25.2	0	0
2	32	Female	I	7	0.24	2.9	7.7	2	2
3	45	Female	V	4	1.34	3.7	8.2	2	4
4	27	Male	V	12	6.18	2.6	5.6	0	1
5	31	Female	V	3	0.59	4.0	5.5	2	0

Abbreviations: ITAS‐A, Indian Takayasu clinical activity; NIH, National institutes of Health; TAK, Takayasu arteritis; TNF, tumor necrosis factor.

### 3.2 Diversity index difference

#### 3.2.1 D50 index

**Figure 1:**
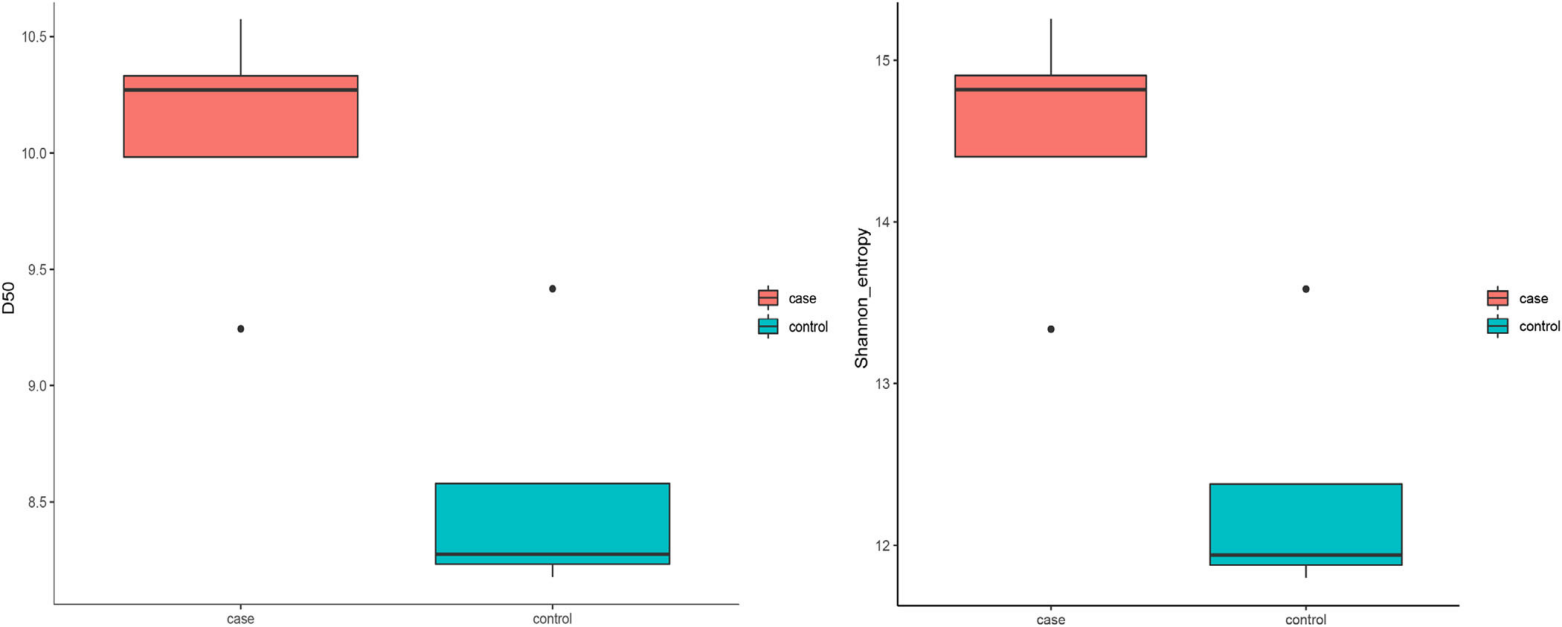
Box plot of diversity index of T cell α chain CDR3 in TAK group and control group. TAK, Takayasu arteritis.

In this study, we used D50 to analyze the diversity of CDR3 in two groups of samples. The results showed that the D50 index of the TAK group was significantly higher than that of the control group (Figure 1). It shows that patients’ CDR3 diversity of T cell was higher than in the control group.

#### 3.2.2 Shannon entropy

The Shannon entropy of the TAK group was significantly higher than that of the control group, indicating that the CDR3 diversity of the TAK group was higher (Figure 1).

### 3.3 Principal component analysis

**Figure 2:**
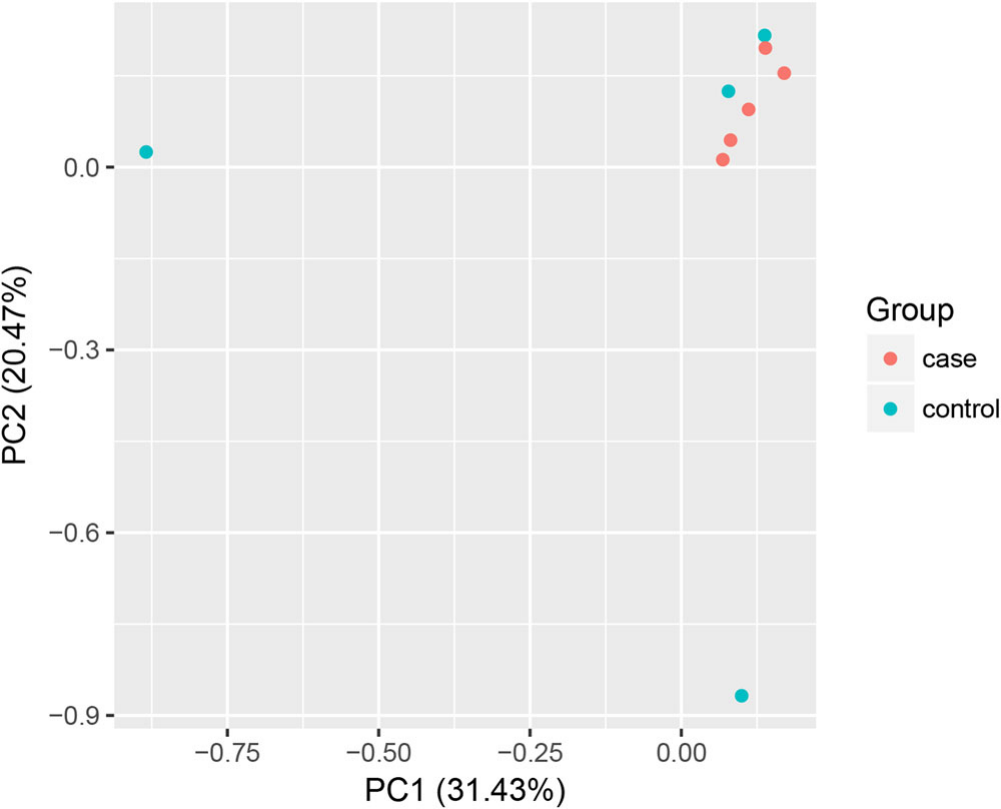
Scatter plot of principal component analysis of T cell α chain CDR3 in TAK group and control group. TAK, Takayasu arteritis.

A two‐dimensional scatter plot is used to judge the heterogeneity of samples in this study. The results showed that the distribution of five samples in the TAK group was close, which indicated that the samples were of good quality (Figure 2).

### 3.4 Gene frequency distribution

We define clones with a frequency greater than 0.1% as high expanded clones (HEC). Mann–Whitney *U* test showed that there was no significant difference in the HEC ratio between the TAK group and the control group. Further analysis of genes with a frequency greater than 0.5% showed that although the proportion of controls was higher than that of patients, there was no significant difference between the two groups.

### 3.5 Analysis of the frequency of gene fragments

**Figure 3:**
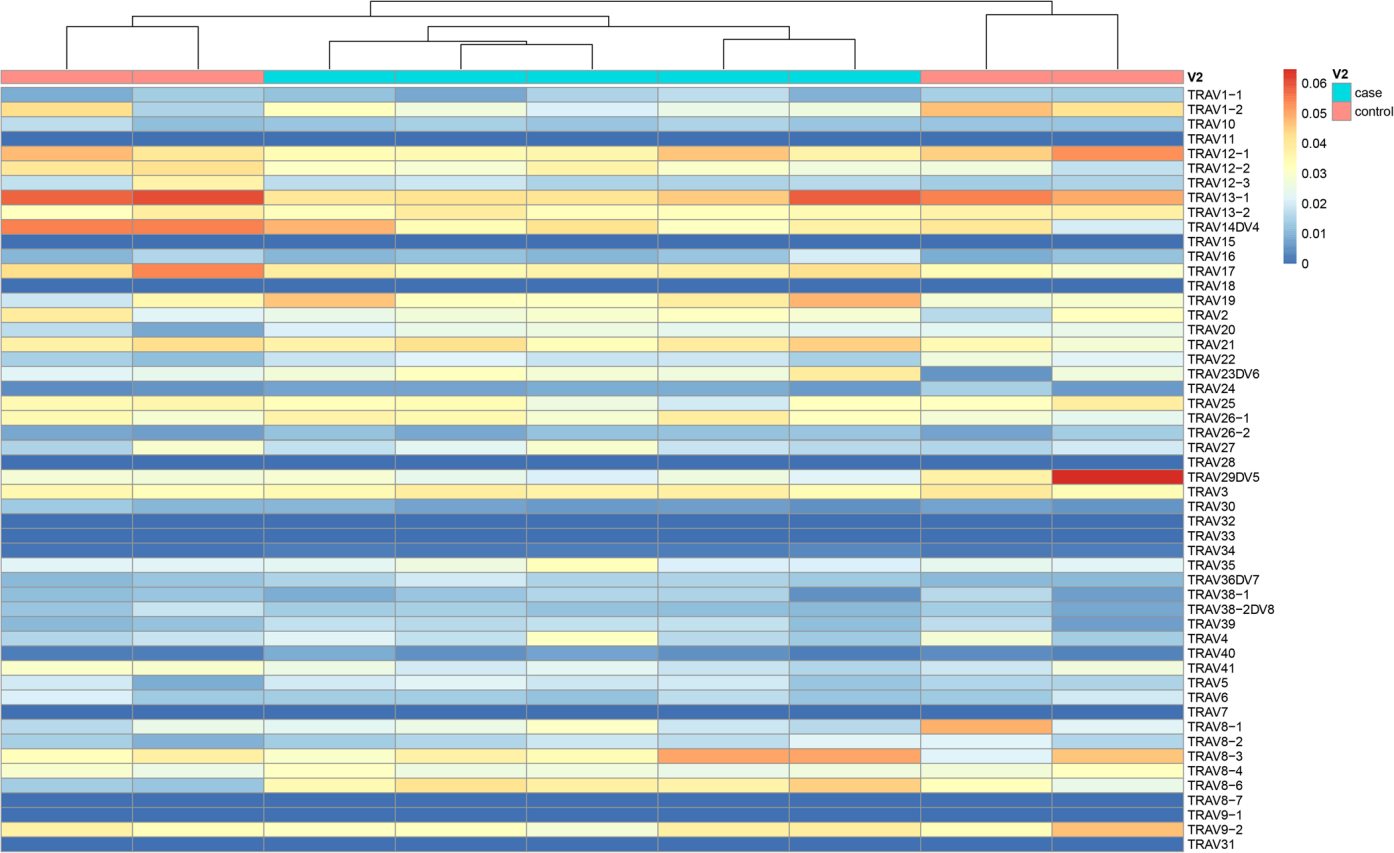
Heat maps of the V region gene frequency of T cell α chain CDR3 in TAK group and control group. TAK, Takayasu arteritis.

We analyzed the frequency of the V region of T cells in the TAK group and the control group. Mann–Whitney *U* test analysis showed three gene fragments with significant differences in frequency between patients and controls. Specifically, the expression of TRAV23DV6, TRAV36DV7, and TRAV8–6 gene fragments in the TAK group was significantly higher than that in the control group (Figure 3).

### 3.6 VJ gene rearrangement difference

Mann–Whitney *U* test showed that there were 196 gene rearrangement fragments between the two groups. Among them, 47 VJ gene rearrangement fragments, such as TRAV12‐1‐TRAJ44, were significantly lower in the TAK group than in the control group. However, the expression of 149 VJ gene rearrangement fragments such as TRAV12‐2‐TRAJ37 in the TAK group was significantly higher than in the control group. Further study found that the three V‐region genes mentioned above also played a pivotal role in VJ rearrangement (Table 2).

**Table 2:** Mann–Whitney *U* test of portion VJ rearrangement genes in TAK group and control group.

Rearrangement gene	TAK group (×10^−4^)	Control group (×10^−4^)	*p* value
TRAV23DV6‐TRAJ10	4.77 (4.32–6.95)	1.79 (1.24–2.09)	.014
TRAV23DV6‐TRAJ14	0.40 (0.37–0.41)	0.12 (0.08–0.21)	.014
TRAV23DV6‐TRAJ3	2.35 (2.00–3.63)	0.18 (0.15–0.31)	.014
TRAV23DV6‐TRAJ31	9.68 (8.20–11.3)	2.54 (2.29–3.57)	.014
TRAV23DV6‐TRAJ37	13.9 (12.9–15.3)	9.17 (6.63–10.3)	.014
TRAV23DV6‐TRAJ5	1.42 (1.35–1.44)	0.29 (0.16–0.42)	.014
TRAV23DV6‐TRAJ7	0.83 (0.80–0.99)	0.11 (0.10–0.17)	.014
TRAV23DV6‐TRAJ8	3.41 (2.97–3.53)	1.33 (0.61–2.11)	.014
TRAV36DV7‐TRAJ11	0.34 (0.05–0.41)	0.02 (0.01–0.03)	.014
TRAV36DV7‐TRAJ12	0.72 (0.44–1.98)	0.02 (0.01–0.04)	.014
TRAV36DV7‐TRAJ20	1.08 (0.88–1.42)	0.06 (0.04–0.23)	.014
TRAV36DV7‐TRAJ29	8.45 (7.14–8.63)	0.87 (0.13–2.03)	.014
TRAV36DV7‐TRAJ36	3.75 (3.61–3.92)	0.20 (0.04–0.73)	.014
TRAV36DV7‐TRAJ4	0.03 (0.03–0.05)	0.02 (0.02–0.02)	.014
TRAV36DV7‐TRAJ46	1.54 (0.63–2.00)	0.01 (0.01–0.16)	.014
TRAV8‐6‐TRAJ11	5.85 (5.54–6.42)	3.90 (3.57–4.23)	.014
TRAV8‐6‐TRAJ13	10.7 (10.6–11.2)	6.17 (5.21–6.79)	.014
TRAV8‐6‐TRAJ21	6.71 (6.40–9.29)	3.30 (2.75–3.80)	.014
TRAV8‐6‐TRAJ23	9.50 (8.51–10.0)	3.74 (1.73–6.03)	.014
TRAV8‐6‐TRAJ3	6.89 (6.47–7.09)	0.81 (0.72–1.32)	.014
TRAV8‐6‐TRAJ32	8.23 (8.08–9.82)	2.43 (1.07–4.58)	.014
TRAV8‐6‐TRAJ37	15.1 (14.2–18.7)	8.42 (6.35–10.9)	.014
TRAV8‐6‐TRAJ41	5.07 (5.06–6.47)	1.17 (0.87–2.18)	.014
TRAV8‐6‐TRAJ47	8.06 (6.34–8.77)	3.28 (1.83–4.57)	.014
TRAV8‐6‐TRAJ8	11.3 (8.22–12.7)	3.50 (2.30–5.14)	.014
TRAV8‐6‐TRAJ46	0.73 (0.57–1.68)	0.007 (0.004–0.01)	.014

Abbreviation: TAK, Takayasu arteritis.

**Figure 4:**
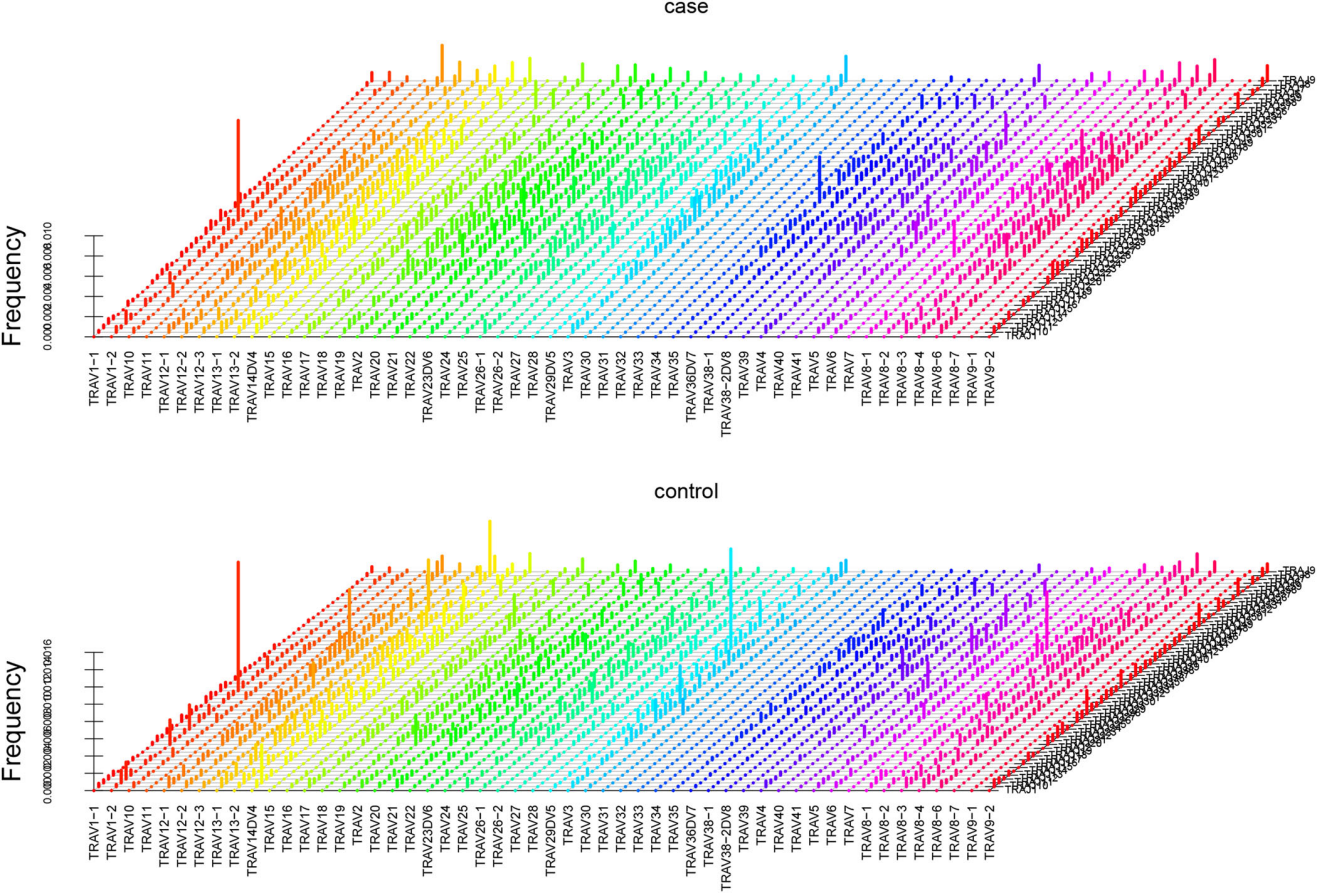
Frequency chart of the VJ rearrangement gene frequency of T cell α chain CDR3 in TAK group and control group. TAK, Takayasu arteritis.

According to the frequency of VJ rearrangement genes, we draw the frequency charts of the TAK group and the control group, respectively, which showed that the TAK group has a unique VJ subfamily (Figure 4).

## 4 DISCUSSION

In this study, we compared the sequencing analysis of the TCR α‐chain CDR3 in blood of patients with TAK and the control group. TCR diversity, V region gene frequency, and VJ rearrangement in patients with TAK significantly differ from those in the control group.

Although the number of cases in this study is small, the PCA shows that each sample is distributed intensively. This study shows that the diversity of TCR in patients is significantly higher than in the control group. This may be due to many different clonal types of T cells being exposed to different antigens after entering the aorta, increasing diversity It is also possible that we chose patients with low activity, and the cloning of T cells in their bodies is not sufficient. In the analysis of gene frequency distribution, although the expression frequency of more than 0.5% of genes in the control group is higher than that in the TAK group, there was no significant difference. It may be caused by the patient's mild condition or after a treatment period.

In the frequency analysis of the V region gene, the frequency of three gene fragments of TRAV23DV6, TRAV36DV7, and TRAV8–6 increased significantly in patients. Suggesting that these genes are probably closely related to TAK. It may be that the stimulation of antigen epitopes on the aorta and its branches of TAK patients changes the CDR3 region of T cells, and T cells combine with antigens through TCR to generate cellular immunity, which leads to inflammatory infiltration. Therefore, T cells with high expression of these CDR3 sequences may be a marker for primary screening of TAK. Preventing the immune function between the corresponding antigen epitopes on the aorta and its branches and T cells may be one of the methods to treat TAK.

Under pathological conditions, Th1 cells and Th17 cells produce inflammatory infiltration and gather in the extracellular membrane. In this case, different cytokines will be produced, which may lead to vascular stenosis, occlusion or aneurysm. Different cytokines will be produced in that case, which may lead to vessel narrowing, occlusion, or aneurysm.^19^

In recent years, many researchers have detected the CDR3 region of TCR in infections, tumors, and other diseases, and there have been many advances in autoimmune diseases.^20^ Some scholars have studied the changes in the CDR3 region of TCR in patients with rheumatoid arthritis (RA) and found that the patients have a unique clonal type.^21^ After that, other scholars used the change of CDR3 as the judgment basis to evaluate the efficacy of abatacept in treating rheumatoid arthritis.^22^ A study analyzed the changes in clinical manifestations and TCR diversity of patients with rheumatoid arthritis after using different drugs.^23^ The results showed that the diversity of TCR was significantly related to the disease activity. In addition, it has been found that in multiple sclerosis, the diversity of patients’ TCR significantly increased after drug treatment.^24^ Because the T cell is one of the key cells in the pathogenesis of TAK, we think that the change of CDR3 may also reflect the change of TAK.

Existing studies have found that HLA‐B*52 has a significant relationship with TAK.^25^ In the HLA locus, it has been proved that amino acid residues 171st and 67th are related to the initiation of TAK. The 67th amino acid can explain the influence of HLA‐B*52 on TAK susceptibility.^26^ In addition, some studies have found that patients with TAK have a specific blood microbiome, and the blood microbiome of patients is related to specific metabolic dysfunction.^27^ Some scholars detected the peripheral blood cells of four TAK patients by RNA sequencing and found significant differences between TXNIP and AREG markers.^28^ Combined with the discovery in this study that the expression of three gene fragments, such as TRAV23DV6, is significantly increased. It is speculated that the gene expression change may be caused by genetic or environmental factors, which further affect the expression of RNA and protein, resulting in inflammatory infiltration.

Scholars have continuously demonstrated that JAK inhibitors have great research value in treating TAK, among which tofacitinib is one of the more promising drugs.^29^ Although there are data at present, the research on DNA sequencing of TAK patients is still an area worth exploring, and future research should focus on sequencing larger samples. In addition, if we can compare the changes in the CDR3 library before and after treatment, we can better describe the CDR3 library of TAK patients. In 2019, a study found significant differences in the TCRβ chain between systemic lupus erythematosus (SLE) and RA patients by sequencing large samples of TCR, so it was recommended as a diagnostic basis. In addition, it is likely to be beneficial to develop targeted therapy and vaccination against SLE and RA.^30^ There is little sequencing research on TAK, and further research on larger samples is needed to reveal its characteristics in the future.

The patients in this study were newly diagnosed TAK patients who were enrolled consecutively over a period of time, thus avoiding the influence of drug factors on the study results. These patients were found to have low disease activity after evaluation of disease activity.

This study still has the following shortcomings: first, the number of samples is small; second, it failed to reflect the dynamic changes of the CDR3 library of patients before and after treatment; third, it is not possible to discuss the CDR3 database in groups according to the patient's condition and treatment.

To sum up, patients with TAK have unique TCR α‐chain clones, among which three gene loci deserve special attention. Due to the small number of cases and other reasons, the above conclusions need to be further discussed in greater and deeper research.

## AUTHOR CONTRIBUTIONS

**All authors**: contributed to the study conception and design. **Na Gao and Lili Pan**: performed material preparation, data collection, and analysis. **Bowen Zha**: wrote the first draft of the manuscript and all authors commented on previous versions of the manuscript. All authors read and approved the final manuscript.

## CONFLICT OF INTEREST STATEMENT

The authors declare no conflicts of interest.

## ETHICS STATEMENT

This study was approved by the Medical Ethics Committee of Beijing Anzhen Hospital (Approval number: 20220.39X), according to the principles of the Declaration of Helsinki, and each subject provided written informed consent for participation in the study. All methods were performed in accordance with relevant guidelines and regulations.

## Data Availability

The data that support the findings of this study are available on request from the corresponding author.
